# Neuroleadership: a new way for happiness management

**DOI:** 10.1057/s41599-023-01642-w

**Published:** 2023-04-04

**Authors:** Raquel Ruiz-Rodríguez, Marta Ortiz-de-Urbina-Criado, Rafael Ravina-Ripoll

**Affiliations:** 1 Universidad Rey Juan Carlos, Madrid, Spain; 2 Universidad de Cádiz, Andalusia, Spain

**Keywords:** Business and management, Business and management

## Abstract

In a post-pandemic era, managers and leaders have a role to enable the changes needed to make workplaces happier and more productive. This paper aims to analyse the role of neuroleadership in the application of happiness management. Two research questions are proposed: what do we know about neuroleadership? and what role can neuroleadership play in happiness management? A mixed methodology is applied. A bibliometric technique is used to identify the main topics studied in neuroleadership. An in-depth analysis and logical reasoning are applied to propose a neuroleadership research roadmap and to present some research pathways for neuroleadership for happiness management. A neuroleadership research roadmap is proposed, considering the approaches, practices/actions, and purposes. It is observed that the consideration of the management of emotions and cognitive processes in the work environment is attracting interest to develop a leadership focused on making better workplaces. A new line of action focused on the management of happiness is emerging. In addition, neuroleadership is presented as a new way of understanding management. Their combined application can mark a before and after in business management, and for that, a proposal of future research lines and questions is developed. This paper presents four future research pathways—perspectives, reasons, adoption and implementation, and results—for studying neuroleadership for happiness management.

## Introduction

Technology has transformed the different facets of human existence and produced rapid changes in society. One interesting effect is that the evolution of technology has greatly supported neuroscience research, leading to very rapid advances in the understanding of the brain and its application in different contexts such as business. In the business context, technological advances are increasingly rapid, especially since the pandemic, where a process of digitalisation of the economy affected both workers and customers (Cuesta-Valiño et al., 2022a; Rangaswamy et al., 2022; Singh and Shaurya, 2021). We live in a society where flexibility is a necessity, and happiness becomes an important variable to understand the effect of technologies on people (citizens, workers, customers).

Society after the pandemic demands a fairer and more sustainable world and economy. The 2030 agenda sets some milestones to achieve sustainable development goals (Keynejad et al., 2021). To achieve these, both workplace and personal well-being are fundamental aspects. In this context, organisations have faced the challenge of improving their management to adapt to the new times. The search for well-being has led to the application of concepts such as happiness management to create better workplaces (Cuesta-Valiño et al., 2022b; Moreno-Ortiz et al., 2022; Ravina-Ripoll et al., 2019a; 2022a). The leaders of companies have the role of making the necessary changes to make the workplaces happier and more productive.

The consideration of emotion management in the workplace and, especially, the cognitive processes associated with it are capturing the interest of companies to develop a leadership focused on decision-making to improve the workplace. Given the current context, in which the phenomenon of the Great Recession and the application of Industry 5.0 are highlighted, there is a lot of attention being paid to the applications and purposes of neuroleadership.

The importance of cognitive neuroscience and leadership is providing an understanding of the biological mechanisms involved in decision-making (Hecht et al., 2013). Furthermore, neuroleadership can help identify skills that cannot be seen but are required for successful leadership (Goldsmith, 2010). For example, two former high-level Gallup leaders, Marcus Buckingham, and Curt Coffman used neuroscience to explain why people are more likely to succeed when managers help them work on their strengths rather than weaknesses (Buckingham and Coffman, 1999). Thus, the interest in the study of neuroleadership is growing. Researchers can produce better-informed theories and leadership patterns by investigating the neurological basis of behaviour (Waldman et al., 2011).

However, the literature studying leadership and happiness is still in its early stages. Different leadership effects and their types on worker well-being and job happiness have been analysed (e.g., Salas-Vallina and Alegre, 2018; Salas-Vallina et al., 2017; Salas-Vallina et al., 2020; Semedo et al., 2017; Setiawan et al., 2020; Wang and Hackett, 2022). Changes in managers’ leadership styles can help improve happiness and job satisfaction (Ahmadiyan et al., 2022). Therefore, the topic of happiness in the workplace can help apply leadership from a different perspective (Alahbabi and Al-shami, 2021).

This paper aims to analyse the role of neuroleadership in the application of happiness management in companies. To this end, the following research questions are presented:

Research question 1: What do we know about neuroleadership?

Research question 2: What role can neuroleadership play in happiness management?

To answer the first question, we identify the main themes in the neuroleadership literature and propose a research roadmap. For the second question, we analyse the applications of neuroleadership for happiness management and propose some future research pathways.

To achieve these objectives, the second section analyses the literature reviews on this topic are analysed. A bibliometric technique known as co-word analysis is used to identify the main topics studied and to show the changes that have occurred between the pre-pandemic (1992–2019) and pandemic (2020–2022) periods. Based on these results, a research roadmap is proposed. The next section explains four future research pathways for neuroleadership and happiness management Finally, the fourth section contains conclusions, contributions, limitations, and future lines of research.

This paper presents an integrative review to generate orderly knowledge spaces due to “they place boundaries around an existing area of research with the purpose of offering an organised display of what is available and building a platform for further research” (Patriotta, 2020, p. 1274).

This paper complements and enriches the previous literature in several ways. Firstly, no previous works have analysed the literature about neuroleadership through 2022. This extension of the time horizon has allowed us to consider the inflection point in 2020, a time of major changes with a global scope that requires consideration. Secondly, in that paper, a bibliometric technique is applied to analyse the evolution of the research line. Thirdly, an interesting new research line—neuroleadership for happiness management—is identified as well as four ways to guide future research are presented.

## What do we know about neuroleadership?

Neuroleadership aims to strengthen the field of leadership with the support of neuroscience findings (Gocen, 2021). The concept of neuroleadership was developed under the leadership of David Rock (Ghadiri et al., 2013). Ochsner and Lieberman (2001) define social cognitive neuroscience as a subfield of neuroscience that attempts to understand human interactions in social, cognitive, and neural aspects. Neurological variables can provide more comprehensive information about “what leaders do and why” (Waldman et al., 2011). Neuroleadership can be seen as an applied field of social cognitive neuroscience that aims to analyse and understand the behaviour of leaders (Badenhorst, 2015; Liu et al., 2015).

Neuroleadership is a topic that has captured the attention of academics in recent years. However, few literature review studies have been conducted on this topic, and those have done partial analyses of the literature. Kuhlmann and Kadgien (2018) review the emerging themes in the neuroleadership literature and present some of the theoretical gaps and possible applications and their consequences. Issac and Issac (2020) perform a biblio-morphological analysis with VOSviewer software to analyse the interdisciplinary interactions between neuroscience and leadership studies. They use the Scopus and Web of Science databases to review the literature combining neuroscience and leadership research in the period 1994–2018. Issac and Issac (2020) present a mapping based on text data, index keywords, main authors who are the pioneers in the area, and countries, which are the leaders in the field of neuroscience and leadership studies. Morphological analysis is executed by dissecting the topic into various dimensions (Genre, Activities, Attributes, Inter-disciplinary, Perspectives, and Efficacy) cross-matched in a cross-consistency matrix that reflects the 174 research gaps that exist in this particular area (Issac and Issac, 2020). Gocen (2021) conducts a systematic review of the literature on the educational and managerial implications of neuroleadership and analyses 44 studies published between January 2010 and May 2020.

To complete the previous literature reviews, we propose a literature review that combines the two areas—neuroscience and leadership—and includes the term neuroleadership. In addition, the latest years are included, and the analysis is carried out up to 2022, presenting an analysis for all years and sub-periods.

## Methodology

To identify the main themes/aspects of neuroleadership that have been studied, a co-word analysis was used in conjunction with the SciMat programme (Cobo et al., 2012). The Scopus database was used, and the fields Title, Keywords, and Abstract were searched. The query used on April 21, 2022, is (TITLE-ABS-KEY (neuroleadership) OR TITLE-ABS-KEY (neuroscience AND leadership). The search returned 412 documents. Keywords were filtered, so the plurals and singulars of the same words were automatically grouped, and words were manually grouped by common synonyms, leaving a total of 818 words or groups of words.

To identify the knowledge structure of the topic under study, the results of the co-word analysis are presented. Callon et al. (1991) propose the classification of each thematic network into one of the following groups: well-developed and isolated themes (upper left quadrant), emerging or disappearing themes (lower left quadrant), basic and transversal themes (lower right quadrant) and driving themes (upper right quadrant), taking into account measures of centrality and density and thus creating a strategic diagram.

## Results

### Knowledge structure of the neuroleadership literature

**Fig. 1:**
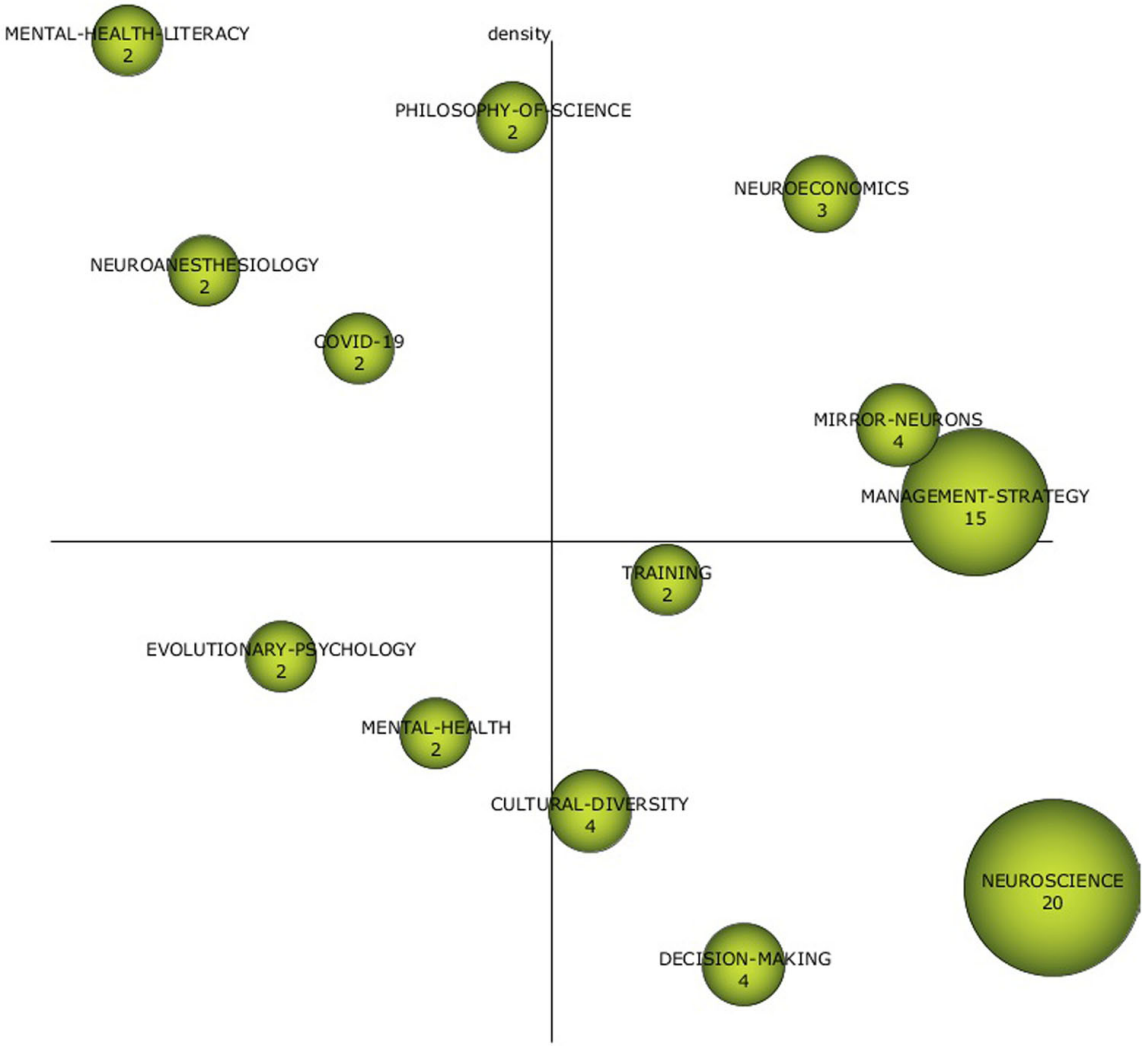
Strategic diagram (1992–2022). Results from SciMat, diagram composed of themes by number of documents for all the period (1992–2022).

**Fig. 2:**
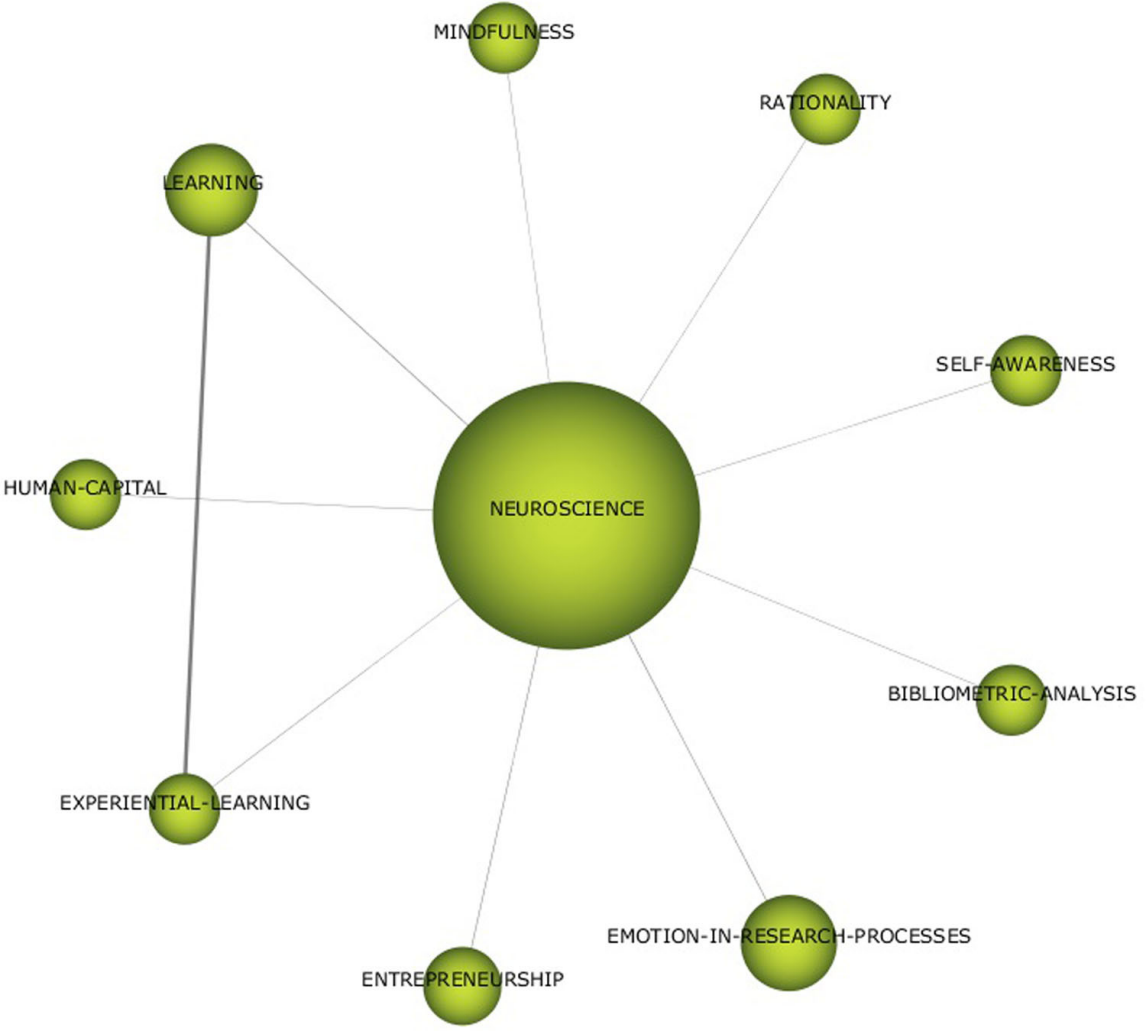
Thematic network for NEUROSCIENCE (1992–2022). Results from SciMat, cluster network for Neuroscience (1992–2022).

**Fig. 3:**
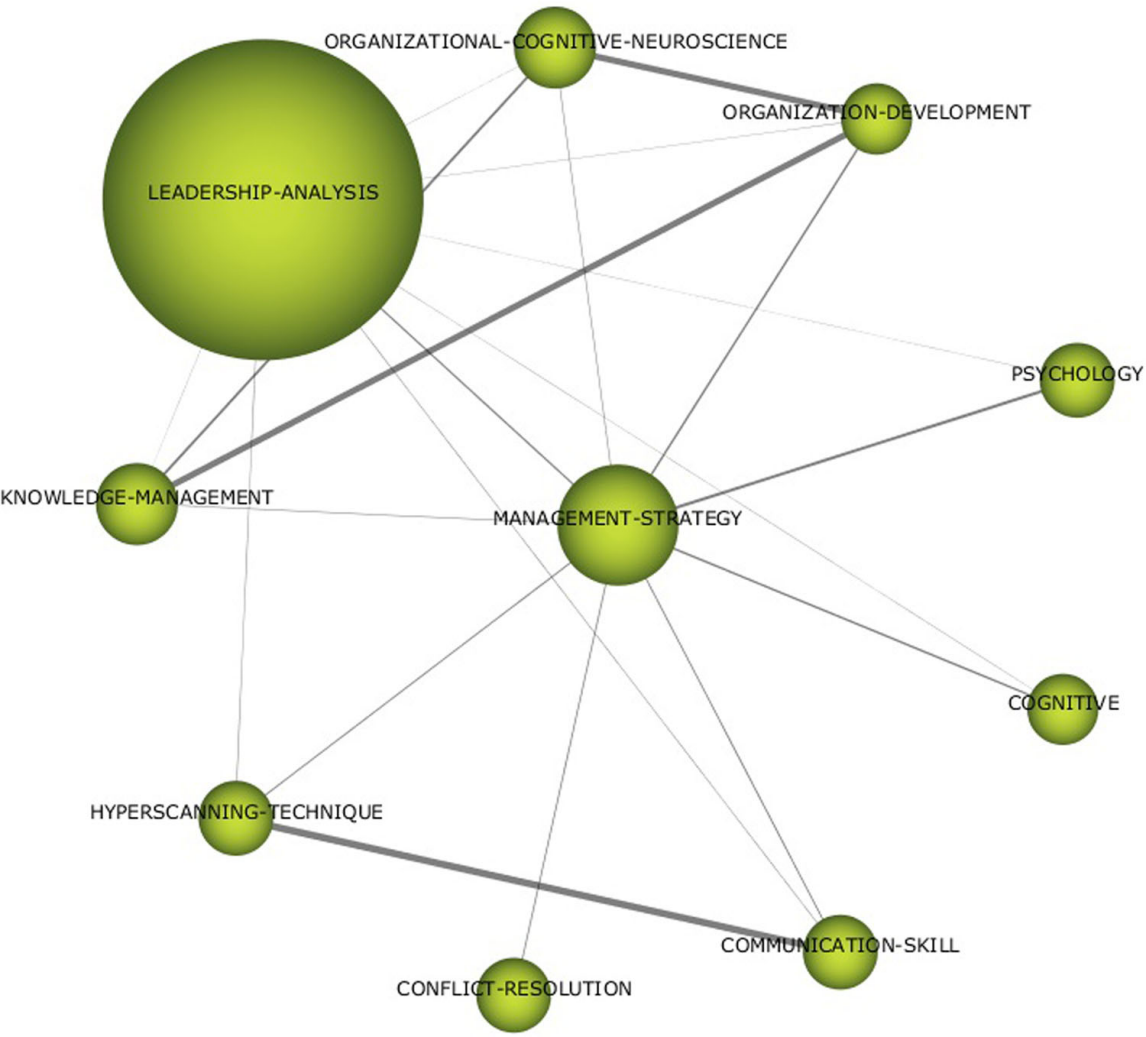
Thematic network for STRATEGIC MANAGEMENT (1992–2022). Results from SciMat, cluster network for Strategic management (1992–2022).

Figure 1 presents the strategic diagram obtained from this analysis for the whole period (1992–2022). The most influential topics are neuroscience and strategic management. These two topics provide the theoretical and academic foundation for the literature on neuro-leadership. The leadership part has mainly been developed from the management literature, and the concept of neuro-leadership has arisen from the application of neuroscience to leadership. It is interesting to analyse in detail the networks of both topics (Figs. 2 and 3).

As we can see in Fig. 2, the aspects that have been applied from neuroscience to develop the research line on neuroleadership stand out. The use of bibliometric analysis and learning through experiments is highlighted. The application of neuroscience has been carried out mainly from a rational perspective, but in its application to the field of human capital, the study of emotional and self-awareness aspects stands out. The consideration of mindfulness that has been gradually introduced in companies and is becoming more widespread is also interesting.

As shown in Fig. 3, the aspects of strategic management applied to develop the neuro-leadership research field are highlighted. The context of leadership analysis and knowledge management stands out. Its application has been mainly based on the development of organisational behaviour and psychology, especially in the study of issues such as conflict resolution, cognitive aspects, and communication skills.

**Fig. 4:**
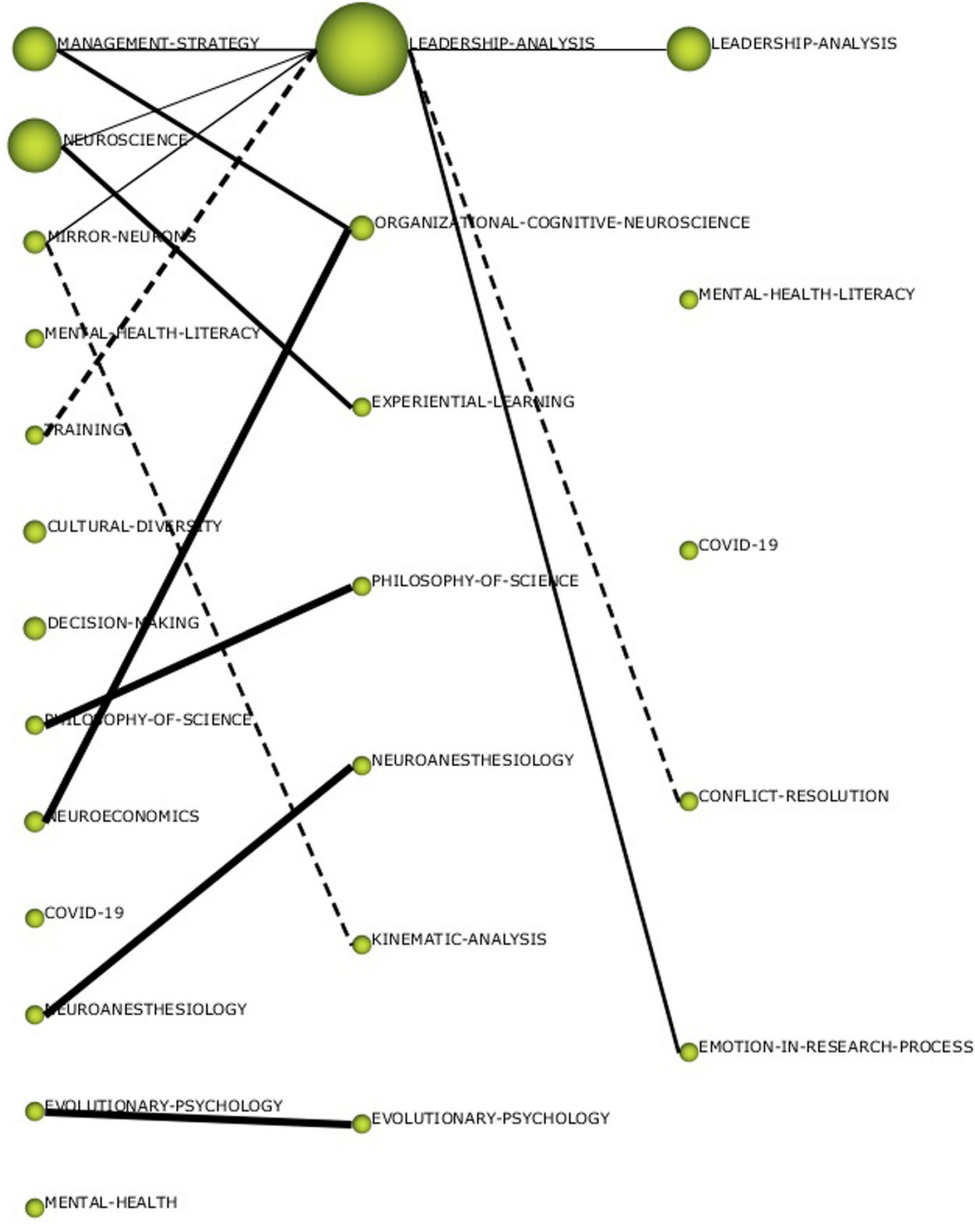
Thematic evolution of the research field (number of documents). Results from SciMat. The first figure is for all period (1992–2022), the second for pre-pandemic (1992–2019) and the last one for pandemic (2020–2022).

To better understand the evolution of the knowledge structure and its characteristics in recent years, the pre-pandemic (1992–2019) and pandemic (2020–2022) periods have also been analysed separately (Fig. 4). Sternitzke and Bergmann (2009) inclusion index is used as a measure to determine the level of similarity between two thematic networks with elements in common during consecutive periods. From this index, a graph is constructed that represents each theme with circles and the lines between the thematic networks show the inclusion index. The size of the circle represents the number of documents in each network. The solid line appears when the central node of one or both networks is included in the other network, while the dotted line appears when the networks share elements that are not central nodes.

Based on the themes identified in Fig. 4, a model of analysis has been proposed (Table 1). As can be seen, the aspects developed in the literature can be grouped into four large blocks: the context (where it is framed), the frameworks/approaches (from which theoretical aspects this literature is developed), actions/practices (how and what has been studied), and the purpose (what for).

**Table 1:** Thematic analysis model by periods.

	Full period (1992–2022)	1992–2019	2020–2022
Context	Neuroeconomics	Philosophy-of-science	Covid-19
	Philosophy-of-science		
	Covid-19		
Frameworks/approaches	Management-strategy	Organisational-cognitive-neuroscience	Mental-health-literacy
	Neuroscience	Kinematic-analysis	
	Mental-health-literacy		
	Evolutionary-psychology	Evolutionary-psychology	
Actions/practices	Training	Experiential-learning	Emotion-in-research-processes
	Cultural-diversity		
Purposes	Decision-making	Leadership-analysis	Leadership-analysis
			Conflict-resolution

Source: Own elaboration.

The context in which the literature on this topic has developed is from the philosophy of science and the application of neuroscience to economics. The effect that the pandemic has had on these studies is also noteworthy. As expected, the theoretical foundations on which previous studies are based come from the field of health sciences, especially neuroscience, mental health, and behavioural psychology. The purpose of studying the topic of neuroleadership is to see how it can be used for better leadership, conflict resolution, and decision-making. For this purpose, among other things, experiential learning, training, the emotional processes involved, and the role of cultural diversity in its application have been analysed.

In addition to the thematic grouping, it is interesting to look in more detail at the strategy diagrams for the two periods.

**Fig. 5:**
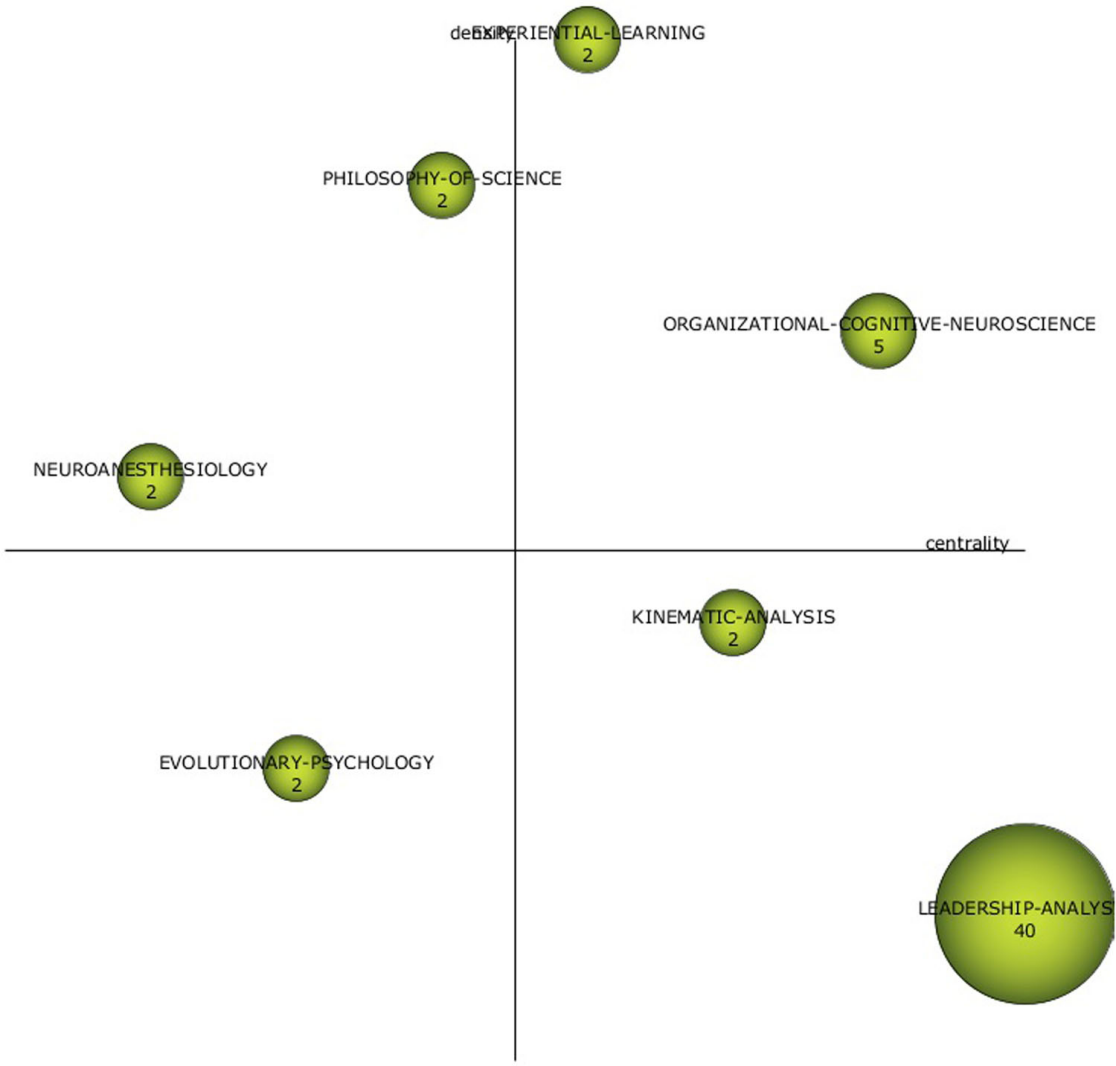
Strategy diagrams by pre-pandemic (1992–2019) period. Results from SciMat, diagram composed of themes by number of documents for (1992–2019).

**Fig. 6:**
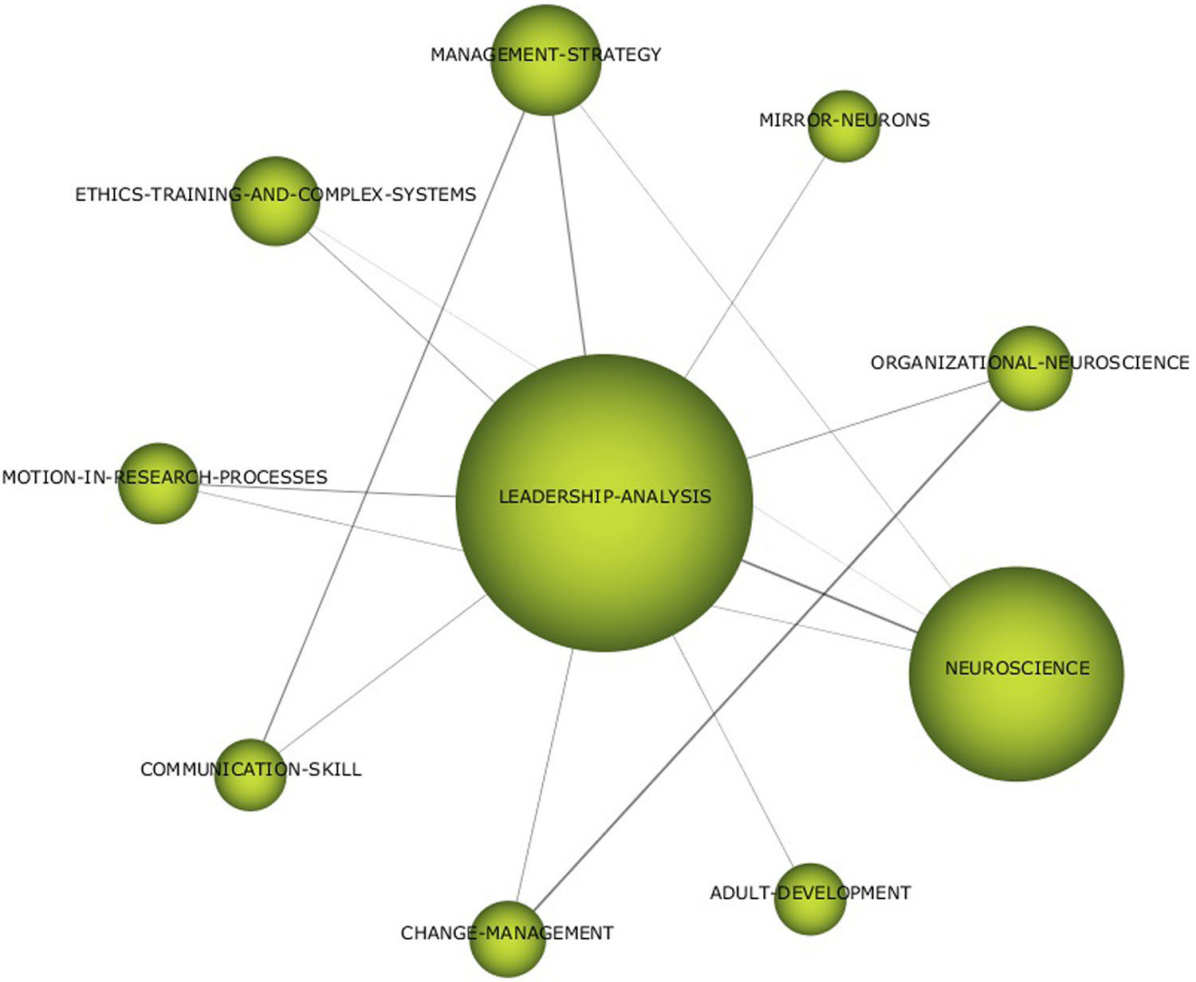
Thematic networks for LEADEARSHIP ANALYSIS (1992–2019). Results from SciMat, cluster network for leadearship analysis (1992–2019).

**Fig. 7:**
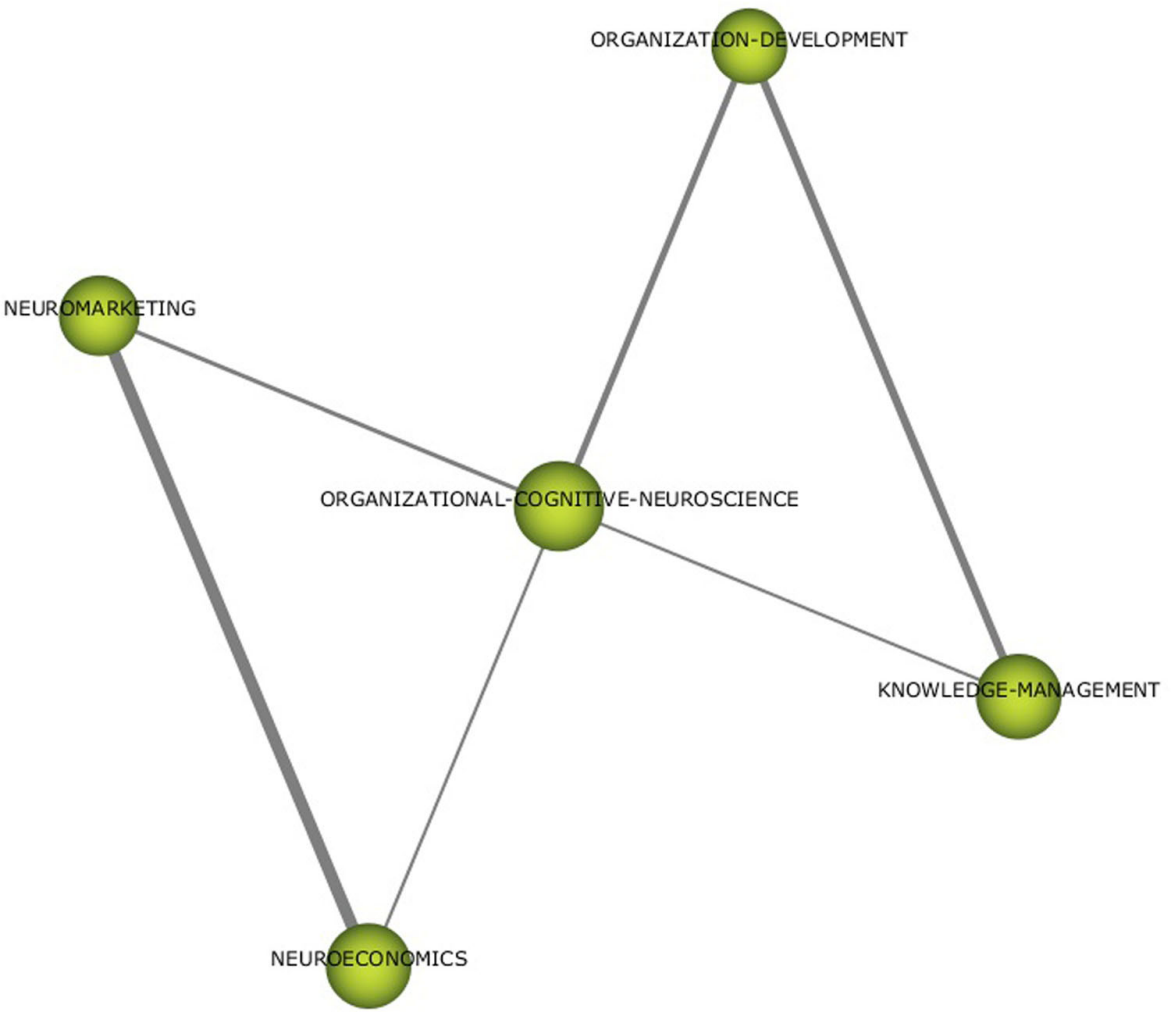
Thematic networks for ORGANISATIONAL-COGNITIVE-NEUROSCIENCE (1992–2019). Results from SciMat, cluster network for organisational-cognitive-neuroscience (1992–2019).

For the pre-pandemic period (Fig. 5), the motor themes are ORGANISATIONAL-COGNITIVE-NEUROSCIENCE and EXPERIENTIAL LEARNING. As basic topics, LEADERSHIP ANALYSIS and KINEMATIC ANALYSIS stand out. While the well-developed topics with the PHILOSOPHY OF SCIENCE and NEUROANESTHESIOLOGY. Finally, the emerging theme is the application of EVOLUTIONARY PSYCHOLOGY. Of all of them, those shown in Figs. 6 and 7 stand out for their network of relationships.

In the case of LEADERSHIP ANALYSIS (Fig. 6), the literature developed up to 2019 was based on neuroscience and its application in organisations. From the paradigm of business management strategies and change management, the literature has focused on topics such as emotional processes, communication skills, and issues related to ethics and complex systems. In the ORGANISATIONAL-COGNITIVE-NEUROSCIENCE network (Fig. 7), the topics considered as frameworks for analysis are neuroeconomics, neuromarketing, knowledge management, and organisational behaviour.

**Fig. 8:**
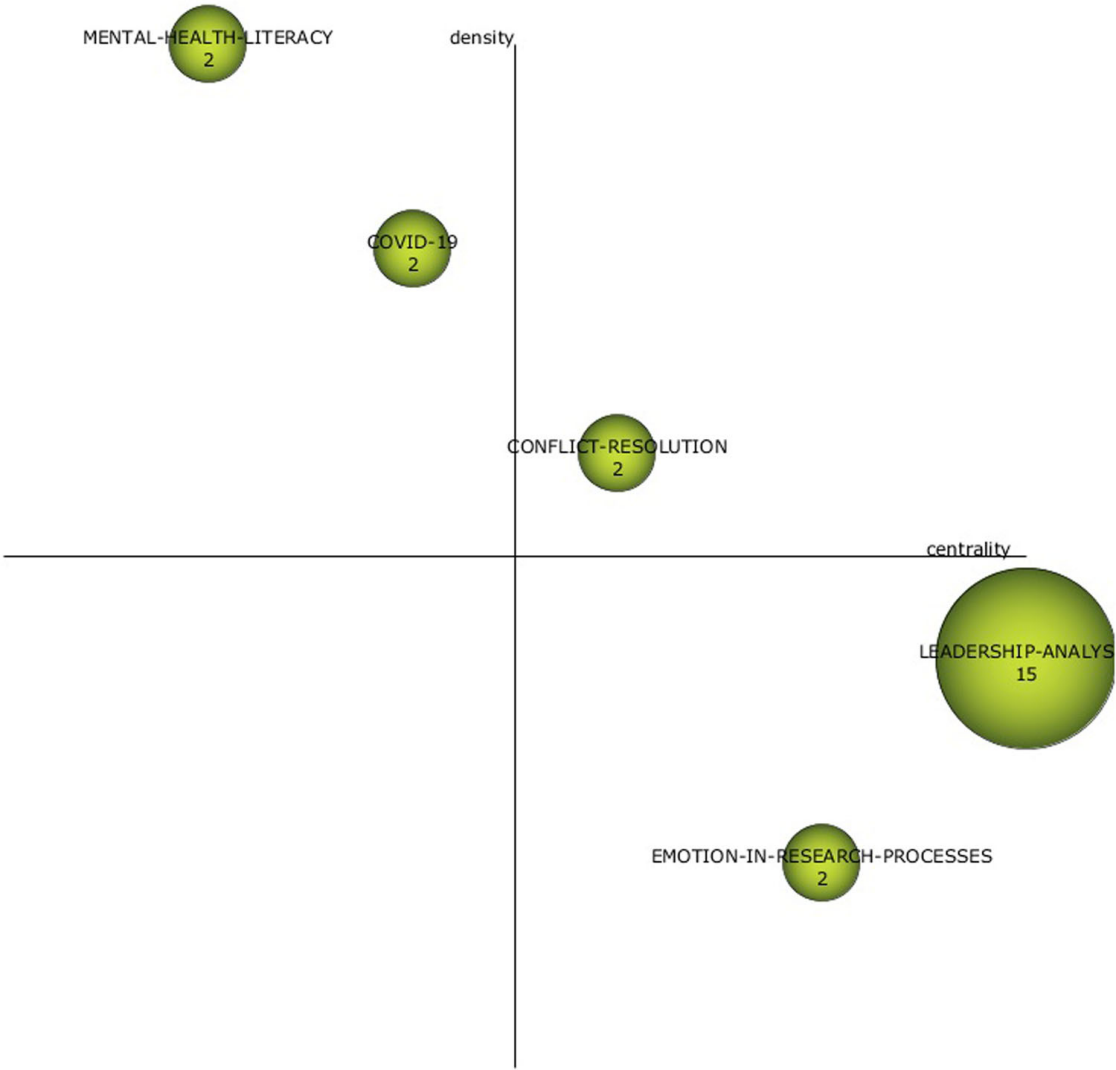
Strategy diagrams by pandemic (2020–2022) period. Results from SciMat, diagram composed of themes by number of documents for (2020–2022).

**Fig. 9:**
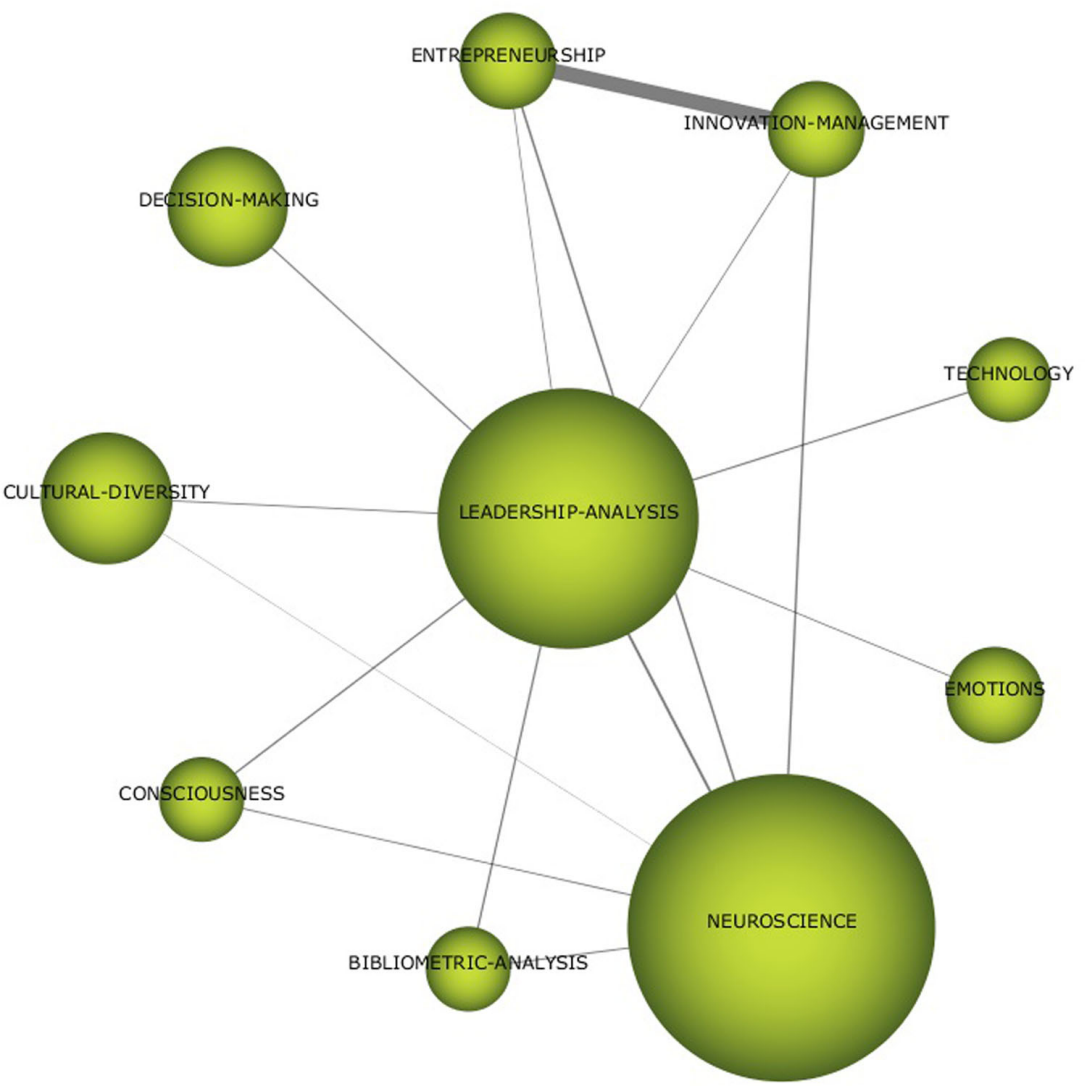
Thematic network for LEADEARSHIP ANALYSIS (2020–2022). Results from SciMat, cluster network for leadearship analysis (2020–2022).

For the last years (Fig. 8), the motor theme is CONFLICT RESOLUTION with those themes that stood out in the previous period (ORGANISATIONAL-COGNITIVE-NEUROSCIENCE and LEADERSHIP ANALYSIS) becoming basic themes. As well-developed themes, we find the literature on mental health and COVID. Of these, LEADERSHIP-ANALYSIS stands out in the COVID era for its network of relationships (Fig. 9).

For LEADERSHIP ANALYSIS (Fig. 9), in recent years, the literature has taken neuroscience as its basis and has become more prominent in conducting bibliometric analyses. It can be seen in this network that the topic is now much more focused on analysing the means and ends of neuroleadership. The consideration of emotions and consciousness as skills to be developed and the application of technology make it possible to develop leadership focused on decision-making, business innovation, and entrepreneurship, without forgetting the effect that cultural diversity can have on its application (Cuesta-Valiño et al., 2021a).

### Neuroleadership research roadmap

Based on the results obtained in the previous section, a process of analysis and reflection has been carried out to establish a research roadmap to define emerging and future issues (Table 2).

**Table 2:** A neuroleadership research roadmap.

	Well-developed topics	Basic topics	Motor topics	Emerging topics	Future topics
Approaches (Where?)	Philosophy-of-science Mental-health-literacy COVID-19	Neuroscience	Management-strategy Neuroeconomics	Evolutionary-psychology Mental-health	Post-pandemic Great Resignation Industry 5.0
Practices/actions (For whom?/how?)		Training Cultural-diversity Emotion-in-research-processes	Experiential-learning		Stakeholders’ analysis Cognitive processes Happiness management
Purposes (For what?)		Leadership-analysis Decision-making	Conflict-resolution		KPIs developed Organisational success Value creation

Source: own elaboration.

Given the current context, which highlights the phenomenon of the Great Resignation and the implementation of Industry 5.0, much attention is being paid to the applications and purposes of neuroleadership. Consideration of the management of emotions in the work environment and, in particular, the associated cognitive processes are attracting interest in companies to develop leadership focused on decision-making to improve workplaces. Happiness management can be a good way to improve the work climate.

On the other hand, it is important to know the impact of the application of neuroleadership in companies. The issues that have been most studied are those related to its application in decision-making and conflict resolution, but other aspects would be interesting to study, such as its effect on value creation and organisational success. For this, it is necessary to have some outcome measures, so the identification of KPIs may be an interesting future line of research.

Among future topics, we would like to highlight the interest in studying the application of neuroleadership in happiness management. In a post-pandemic context, the pursuit of happiness is a good way to focus on the changes that both individuals and the organisation are making or will have to implement. In the context of the workplace, a new line of action is emerging that focuses on happiness management, which can help companies to be more successful and create more economic and social value. For its part, neuroleadership is also presented as a new way of understanding business management. Their joint application can mark a before and after in the management of companies, an issue that we will reflect on in the following section.

## What role can neuroleadership play in happiness management?

### Neuroleadership for happiness management

From the beginning of the 21st century to the present day, a significant body of researchers has been devoted to exploring happiness at work as one of the most important means for corporate governance to increase productivity, innovation, competitiveness, or the creative skills of their internal customers in today’s digital society (e.g., Aboramadan and Kundi, 2022; Galván Vela et al., 2022; Srivastava et al., 2022).

Based on this phenomenon, and according to the information consulted in the Scopus and Wos databases, we can see that this rich literature has served, among many other academic aspects, for more than 20 articles to emerge between 2009 and 2022 for the search of academic text titles using the following search string “happiness management”. In the first study of this series of scientific papers on this particular type of organisational culture, the authors show that a positive working climate can be fostered within companies using management models that stimulate the happiness of their employees in the day-to-day performance of their professional activities (Gao et al., 2009). In recent years, some scientific publications began to emerge that empirically demonstrate the significant interactions of happiness management on the following dimensions: work climate, commitment, social marketing, loyalty, transformational leadership, corporate image, and organisational justice (e.g., Cuesta-Valiño et al., 2021b; 2023; Elías-Zambrano et al., 2023; Núñez-Barriopedro et al., 2021; Rosenberg, 2010; Sánchez-Vázquez and Sánchez-Ordóñez, 2019).

Undoubtedly, the proliferation of this research contributed to the fact that, in 2019, the academic world defined for the first time this attractive corporate culture as a multicultural management model aimed at encouraging the professional activity of its internal customers through the virtuous circle of corporate happiness (Ravina-Ripoll et al., 2019b). The biometric analysis carried out by the authors of this article on the philosophy of happiness management reveals that some of its specialists have focused on exploring what kind of leadership is required by a given organisational culture to foster happiness at work in the post-Covid-19 era (Jiménez-Marín et al., 2021). Currently, these social science studies are in their embryonic stage of scientific production (Jambrino-Maldonado et al., 2022).

An analysis of the main papers found in Scopus and WoS on leadership and happiness has been carried out to establish the framework of study for the application of the new form of leadership, neuroleadership in the management of happiness. Previous literature in recent years has analysed different effects of leadership and its types on employee well-being and happiness in the workplace. Positive effects on happiness at work have been found for different types of leadership such as virtuous leadership (Wang and Hackett, 2022), inspirational leadership (Salas-Vallina et al., 2020), altruistic leadership (Salas-Vallina and Alegre, 2018), authentic leadership (Semedo et al., 2017; 2019), spiritual leadership (Srivastava et al., 2022), ethical leadership (Yang, 2014), or servant leadership (Alahbabi and Al-shami, 2021; Salas-Vallina and Fernández-Guerrero, 2018). But one of the most studied is transformational leadership, which has been considered an antecedent of happiness at work (Salas-Vallina et al., 2017; Setiawan et al., 2020). Other authors have identified the influence of happiness at work as a mediator of the relationship between transformational leadership and affective commitment (Abdullah et al., 2017). From the opposite approach, destructive leadership is observed to have negative effects on employees’ happiness and psychological detachment (Syed et al., 2022). We can find some studies on the relationships between self-leadership, work stress, and happiness (Jwa et al., 2018). Other aspects analysed have been the effects of changes in leadership models. Authors such as Ahmadiyan et al. (2022) find that changes in managers’ leadership style significantly affect organisational climate and can help improve happiness and job satisfaction.

Most of these works make empirical studies of structural equations, and one stands out that starts from other well-being models such as the PERMA model—Positive Emotion, Engagement, Relationships, Meaning, and Fulfilment—and the SHANARRI wheel—Safe, Healthy, Successful, Nurtured, Active, Respected, Responsible, Included—(Peill, 2022). Human resources with high levels of happiness will affect productivity and the fluidity of tasks performed. Some authors have found that leaders who have the skills to listen and think about employees’ career paths positively influence their employees’ happiness (Isa et al., 2019).

Not surprisingly, there is currently no clear definition of the concept of happy leadership in the discipline of management, leadership, and decision-making. With other multiple aspects, this fact hinders the development of multi-theoretical and practical studies of the leadership-happiness management construct. Recent literature shows that both elements have a positive impact on the competitiveness and sustainability of companies in the medium and long term (Ravina-Ripoll et al., 2022b). In this sense, it is interesting to undertake future research that explores whether neuroleadership is a solid predictor of corporate happiness in ecosystems that enjoy the culture of happiness management (Rando-Cueto et al., 2023). This type of leadership is understood as an asset that can foster organisational happiness through emotional intelligence, employee empowerment, positive emotions, and resilience (Grunwald, 2021; Pittman, 2020).

### Future research pathways for neuroleadership and happiness management

The issue of happiness in the workplace needs to be properly conceptualised to understand the role of leadership from a different perspective (Alahbabi and Al-shami, 2021). What we observe is that there is little work on leadership and happiness management and that new studies on the effect of neuroleadership on happiness management have not yet been developed.

Considering the interest that the study of neuroleadership and its effects on happiness is arousing, we propose some lines of future research grouped into pathways. On the one hand, it is necessary to analyse this topic from different perspectives, especially from neuroscience and organisational behaviour approaches. Secondly, it is interesting to analyse how to improve the application of neuroleadership in cognitive processes. Thirdly, adoption and implementation should be studied through Stakeholders’ analysis models and Happiness management models. And finally, it is necessary to consider its effects, and to do so, KPIs can be developed, value creation can be analysed and its effect on the success of the organisation can be seen.

#### Pathway 1. Perspectives

Organisational behaviour is a classic topic in business management and specifically in human resources management, which has been adapting, incorporating a systemic, open approach, in which people are human beings endowed with intelligence and skills that must be motivated and driven. However, the current environment characterised by uncertainty, and with phenomena such as the great resignation and industry 5.0, new challenges are being posed to improve organisational behaviour models in organisations. One of these challenges is the application of neuroscience, which can help to obtain more sustainable and productive management models, as well as adapt to the new changing reality. The combination of neuroscience and organisational behaviour can lead to the generation of new knowledge and is useful for analysing the human brain and understanding what lies behind human behaviour. For example, it is possible to analyse how mirror neurons influence adaptive behaviours or how the social learning process is processed unconsciously, generating attitudes that involve automatic and rapid behaviours. Therefore, cognitive neuroscience can explain explicit and implicit attitudes and behaviours that determine decision-making (González-Esteban, 2016). Neuroleadership studies have even advanced to brain-computer interface studies (Massaro, 2015). Neuro-leaders manage their institutions by establishing a management strategy based on brain-based findings. Neuroleadership goes beyond behaviour, beyond what is observed, and aims to discover tools for detecting leaders, improving skills, and detecting factors that are unconsciously affecting behaviour. Future research may consider conducting multi-theoretical analyses of neuroscience and organisational behaviour to offer perspectives from ethics and corporate social responsibility on their role in happiness. Future models may develop elements to alleviate high resignation, burnout, and feelings of individual discomfort and generate happiness at work by incorporating cognitive neuroscience research and the processes that underlie it, generating a management model in which people’s happiness is the central element.

#### Pathway 2. Reasons (Before)

Brain scientists emphasise that it is necessary to pay attention to employees’ behaviour, emotions, and motivational stimuli to achieve the best performance. Future studies can promote neuroleadership and cognitive processes as attributes of innovation, creativity, and entrepreneurship to make employees feel happier in their jobs. Through neuroleadership, management models can be implemented that support and contribute to sustainable and happy workplaces. Following the Sustainable Development Goals, providing a response to COVID-19, the United Nations has created a roadmap to support countries in social and economic recovery. Among the goals, we focus on SDG 8 on decent work and improving living standards. In this new paradigm, applying cognitive neuroscience to organisations can generate the answer and achieve management models based on well-being and happiness, managing to boost cognitive power, and cognitive capacities, and training them such as creativity, innovation, or attention, achieving higher levels of productivity, efficiency, and diversity.

#### Pathway 3. Adoption and implementation (During)

Other aspects in which new studies can be developed are models for applying neuroleadership according to the stakeholder, as there are different interests that, if not well managed, can hinder the attraction and retention of talent.

One aspect that is attracting a lot of attention is the different approaches to work and happiness in the workplace of the new generations (Millennials and Generation Z). In this sense, neuroleadership can offer alternatives and models of cognitive processes appropriate to the needs and objectives of each generation.

All of this can be used to develop and validate general stakeholder analysis models from the happiness management approach that serve to increase the work motivation and psychosocial well-being of the various stakeholders. What management models will robotisation and the battle of the generations demand? But in addition to theoretical models, the challenge is to provide empirical evidence of the competitive advantage achieved by implementing the culture of happiness management in neuroleadership models applied to stakeholder management.

#### Pathway 4. Results (After)

Additionally, it would be valuable for future studies to examine the impact of neuroleadership on employee happiness and overall organisational success.

Considering the future ways, several research questions emerge:

- Can neuroleadership practices and principles become standard tools in management to promote workplace happiness?
- Are there opportunities to develop management models centred on the principles of happiness management with the integration of neuroleadership?
- How does the application of neuroleadership affect job satisfaction and happiness of internal and external customers?
- How can technological advances in the application of neuroleadership to happiness management affect the internal and external image experience?

## Conclusions

In a post-pandemic era, managers and leaders have a role to play in enabling the changes needed to make workplaces happier and more productive. In that context, to answer the first research question—what do we know about neuroleadership?—a neuroleadership research roadmap is proposed, considering the approaches (where?), practices/actions (for whom?/how?), and purposes (for what?).

These results help to synthesise the main studied themes of neuroleadership and have allowed us to present the main findings—highlights—in this line of research:

Highlights 1. Neuroleadership literature has mainly been conducted based on the theoretical approaches of Philosophy-of-Science and Mental-Health-literacy.

Highlights 2. Neuroleadership research can be complemented by management strategy and neuroeconomics approaches to better adapt to the challenges of Industry 5.0.

Highlights 3. Neuroleadership literature has been oriented toward the analysis of training, cultural diversity, emotion-in-research-processes, and experiential learning.

Highlights 4. The main purposes of neuroleadership in companies have been decision-making and conflict resolution.

Highlights 5. Neuroleadership adoption in companies is a challenge, and its research is a developing topic.

Then, some future trends are presented:

Future trend 1. Research on neuroleadership has the challenge of showing the importance of its implementation to adapt companies to Industry 5.0 and mitigate the effects of the Great Resignation.

Future trend 2. Neuroleadership research can be enriched by considering the role of various stakeholders in its implementation.

Future trend 3. Neuroleadership research can delve deeper into the cognitive process’s role in the operational and strategic decision-making of companies.

Future trend 4. Research on neuroleadership can deepen the causes and consequences of its application for happiness management.

Future trend 5. Neuroleadership research can delve deeper into the study of its impact on organisational success, developing measurement indicators and analysing their value creation.

But in today’s dynamic and changing environment, we wonder how the COVID period may have affected this line of research. The last few years have been marked by confinement, personal isolation, and teleworking, which have changed work and social environments rather drastically. It is observed that the consideration of the management of emotions and cognitive processes in the work environment is attracting interest to develop a leadership focused on making better workplaces. A new line of action focused on the management of happiness is emerging. Neuroleadership is presented as a new way of understanding management. For these reasons, we have identified an interesting gap (future trend 4), which is the consideration of happiness management in the new styles of leadership. Their combined application can mark a before and after in business management.

In that line, to answer the second research question—what role can neuroleadership play in happiness management?—this paper presents four future research pathways—perspectives, reasons, adoption and implementation, and results—for studying neuroleadership for happiness management.

Pathway 1. The neuroleadership implementations for happiness management can be based on neuroscience and organisational behaviour approaches.

Pathway 2. To improve happiness management, it is interesting to apply models to improve cognitive processes through neuroleadership.

Pathway 3. To improve happiness management, it is interesting to apply models for the application of neuroleadership according to stakeholders.

Pathway 4. An understanding of the effect of neuroleadership on happiness management requires models to measure value creation and its effect on organisational success.

Academics and professionals are two kinds of readers of this paper due to several theoretical and practical lessons that can be learned. Academics can know the state-of-the-art neuroleadership research and understand how it has evolved in an uncertain environment. The pandemic situation and 2020 constitute a turning point that has led to important changes in leadership and happiness management.

One of the theoretical contributions is the identification of the main themes in neuroleadership research, and the general research roadmap proposed. This roadmap shows some answers to three questions: (1) Where is neuroleadership being analyzed? (2) How is neuroleadership being applied? (3) What is it used for? The third theoretical contribution of this work is that we have identified what we have not explored, that is their role in happiness management and the main future pathways that can be followed to bring them to light.

This research can play an important role in advancing research by synthesising and organising existing knowledge and identifying areas for future investigation. This paper presents an integrative review (Patriotta, 2020) that offers another voice and speaking position for re-inventing and writing new papers about neuroleadership and happiness management. Post et al. (2020) emphasise the importance of articles that can serve several purposes, including helping researchers understand the research topic, discerning important and under-examined areas, connecting research findings from disparate sources to create new perspectives and phenomena, and bridging fragmented areas of research by developing links between established but previously unconnected theoretical perspectives.

Additionally, we cannot forget the importance of connecting the world of academic research with the real business world. Markman (2022) argues that it is important for research to address problems that affect people, businesses, and society to make the world a better place. This paper could help professionals and leaders to understand better how to manage happiness in the organisation. In that context, this paper presents a proposal for better workplaces and happier workers and people.

However, the paper also acknowledges the limitations of the methodology used and calls for further research to expand our understanding of the topic. Future studies could complement the co-word analysis with other bibliometric techniques, such as co-citation analysis, or can also apply complementary methodologies such as content analysis or case studies. Statistical models, such as structural equation models and decision trees, can also be used for studying neuroleadership adoption and its applications for happiness management.

Another interesting topic for future studies is explaining the ethical aspects to be considered when implementing management and leadership practices from a neuroscience approach. In this context, happiness management models can help to implement new forms of leadership from a perspective that considers environmental, ethical, and corporate social responsibility issues. Neuroleadership can be a good tool to develop these models, but they require changes in organisations. It would therefore be interesting to carry out studies on the good practices of companies, which are implementing ethical and sustainable culture and leadership models to promote diversity, innovation, and happiness in the workplace.

## Data Availability

Documents that support the findings of this study can be consulted in the Scopus database by following the search procedure indicated on methodology section.
